# Penetrating injury to the left ventricle caused by attempted suicide—a case report

**DOI:** 10.1093/jscr/rjae159

**Published:** 2024-03-17

**Authors:** Mara Parentic, Eva Podolski, Marin Korda, Borna Katic, Fran Juraj Kajs, Kristina Krzelj, Drazen Belina, Hrvoje Gasparovic, Tomislav Tokic, Zeljko Duric

**Affiliations:** University of Zagreb, School of Medicine, Zagreb 10000, Croatia; University of Zagreb, School of Medicine, Zagreb 10000, Croatia; University of Zagreb, School of Medicine, Zagreb 10000, Croatia; University of Zagreb, School of Medicine, Zagreb 10000, Croatia; University of Zagreb, School of Medicine, Zagreb 10000, Croatia; Department of Cardiac Surgery, University Hospital Center Zagreb, Zagreb 10000, Croatia; Department of Cardiac Surgery, University Hospital Center Zagreb, Zagreb 10000, Croatia; Department of Cardiac Surgery, University Hospital Center Zagreb, Zagreb 10000, Croatia; Department of Cardiac Surgery, University Hospital Center Zagreb, Zagreb 10000, Croatia; Department of Cardiac Surgery, University Hospital Center Zagreb, Zagreb 10000, Croatia

**Keywords:** cardiac injury, stab wound, cardiac tamponade, hemorrhagic shock

## Abstract

Penetrating cardiac injuries are rare but are one of the most urgent emergencies because they require early intervention in order to prevent death. The mortality rate of such injuries, including pre-hospitalization deaths, goes up to 90%. The most commonly injured heart chamber is the right ventricle since it takes over half of the anterior thoracic wall. The left ventricle is injured less often, but these patients usually have worse prognoses and higher mortality rates because such injuries lead to hemodynamic instability faster. We present a unique case of a suicide attempt in which the patient stabbed himself with a knife, penetrated the left ventricle, and survived even though he transected the second diagonal branch of the left anterior descending coronary artery and pulled the knife out of his chest.

## Introduction

Penetrating cardiac injuries are rare, as they comprise ~0.1% of all trauma admissions [1]. Still, they are one of the most urgent emergencies because they require prompt intervention along with a systematic approach in order to prevent lethal outcome [2, 3].

The two main mechanisms of such injuries are either penetrating stab wounds or penetrating gunshot wounds, with the latter being more present in war areas and areas associated with gang activities. Stab wounds most commonly occur as a result of criminal activity or as a self-inflicted injury, either by suicide attempt or accident [3]. We present a unique case of a suicide attempt in which the patient stabbed himself with a kitchen knife, penetrated the left ventricle (LV), and survived even though he pulled the knife out of his chest and transected the second diagonal branch of the left anterior descending artery (LAD).

## Case report

**Figure 1:**
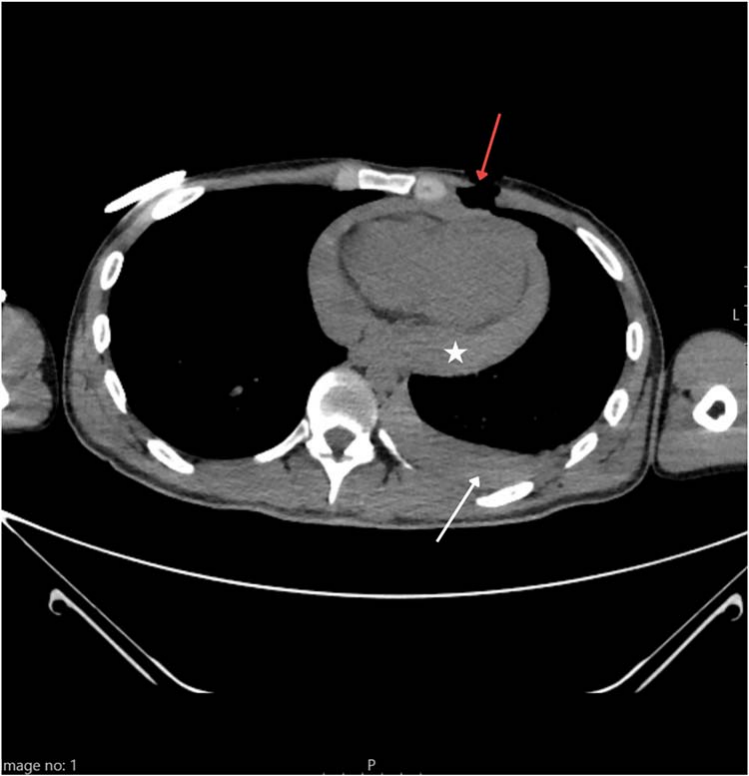
Thoracic CT showing a penetrating wound to the left pectoral area (red arrow) along with hemorrhagic pleural effusion (white arrow) and hemopericardium (star).

A 25-year-old Caucasian male presented to the nearest emergency department following a suicide attempt. He reported trying to commit suicide by stabbing himself with a kitchen knife which he pulled out of his chest after he realized he was not succumbing to death as fast as the pain he felt was intensifying. On admission, he was responsive with a Glasgow Coma Scale of 14, but in hemorrhagic shock with blood pressure of 80/60 mmHg and tachycardia. The patient was immediately admitted to the intensive care unit (ICU) where the initial work-up included physical examination, blood lab tests, chest X-ray, and emergency computed tomography (CT) of the thorax. Norepinephrine and blood transfusions were administered. CT imaging revealed a left pneumothorax measuring a maximum of 41 mm along with a hemothorax of 24 mm, and a hemopericardium that measured 34 mm (Fig. 1). A chest drain was placed into the left pleural cavity, and the patient was transported to the cardiac surgery department of our institution where an emergency surgical procedure was performed.

**Figure 2:**
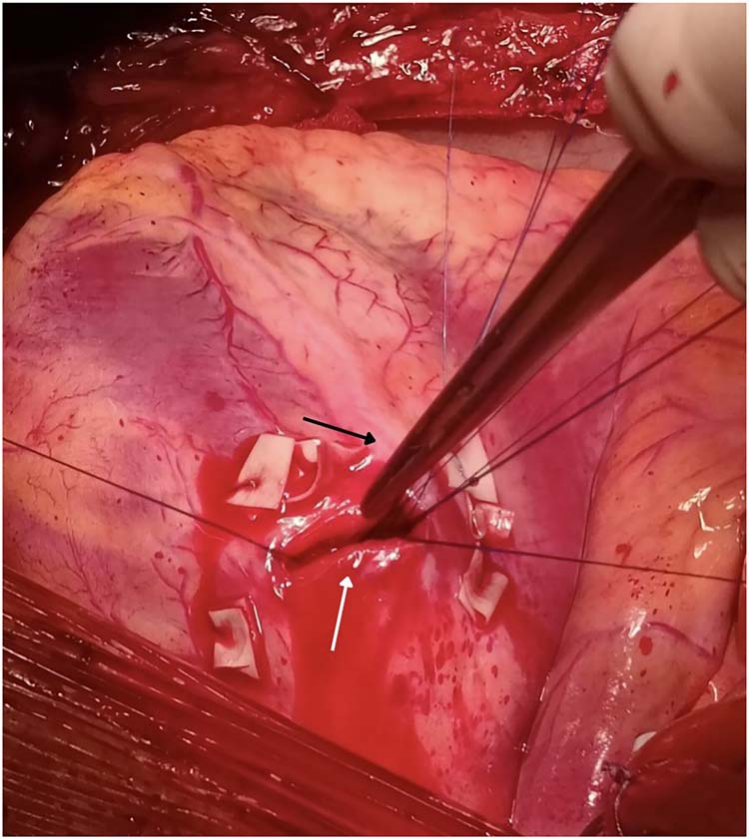
Penetrating wound to the LV measuring 2 cm (white arrow) missing the LAD (black arrow) by 2 mm but transecting the second diagonal branch of the LAD.

Our team performed urgent sternotomy, pericardiotomy and evacuation of hemopericardium, and only then was the full extent of the injury verified – a penetrating wound to the LV which measured ~2 cm and transected the second diagonal branch of LAD (Fig. 2). The knife missed the LAD by ~2 mm.

The heart was then put in cardioplegic arrest and the procedure was carried out with the help of a cardiopulmonary bypass machine. The wound was closed using seven interrupted monofilament sutures with pledgets made from a porcine pericardial patch.

**Figure 3:**
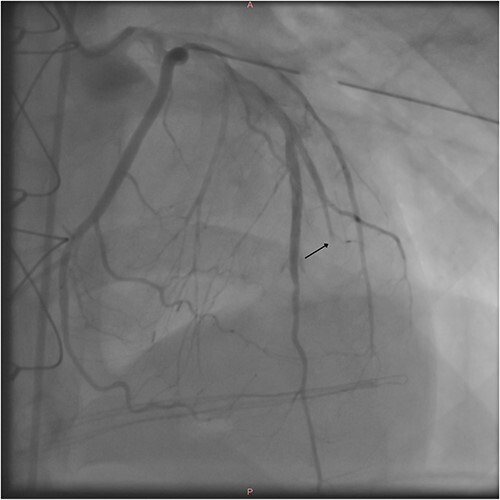
Postoperative coronary angiography shows a transected second diagonal branch of the LAD (arrow).

After the procedure, the patient was hemodynamically stable and transported to the ICU. Postoperative coronary angiogram showed a transected second diagonal branch of LAD (Fig. 3).

The patient’s postoperative echocardiography showed a normal-sized LV with globally preserved systolic function and an ejection fraction of 55%. Following the hospital discharge, the patient was referred to the psychiatric clinic for ongoing care. He recovered well and remained without symptoms on a follow-up.

## Discussion

Because of the location of a heart inside the thoracic cavity, the primary site of a penetrating myocardial injury is the right ventricle (RV) [4, 5]. The LV is injured less often, but these patients usually have worse prognoses and higher mortality rates [6] because such injuries lead to hemodynamic instability faster. RV injury occurs in 43% of the cases, while LV injury accounts for 34% of the injuries. The third most commonly injured heart chamber is the right atrium which accounts for 18% of the injuries [7].

The diagnostic approach for patients with penetrating cardiac injuries must be prompt and systematic. It includes a physical examination, standard blood tests, and echocardiography which is considered the gold standard for diagnosis [3]. If the patient’s condition allows it, a thoracic CT can be done. However, the exact severity of these injuries can only be assessed during the surgical intervention.

Death rates of those with penetrating injuries directly correlate with the time between the injury and the start of surgery [8], and without timely intervention, they go up to 90% [5]. Gervin and Fischer demonstrated the importance of fast intervention by showing that there is a survival advantage for patients who were transported to the trauma center within 9 min, while the mortality rate reached 100% for all those transported to the trauma center after >25 min after the time of the injury [9].

As mentioned before, hemodynamic instability in the left-side chamber injuries correlates with poorer prognosis with reports of survival rates of only 28% - compared with the 79% survival rates of right-ride chamber injuries [6].

In conclusion, penetrating cardiac traumas are rare, and the prognosis of such injuries is poor. In order to reach the best possible outcome for the patient it is of utmost importance to act fast and surgically intervene as soon as possible [5].
